# Incidence of depression in kidney transplant recipients in South Korea: a long-term population-based study

**DOI:** 10.1038/s41598-022-20828-x

**Published:** 2022-10-20

**Authors:** Semin Cho, Sehoon Park, Ji Eun Kim, Mi-yeon Yu, Seon Ha Baek, Kyungdo Han, Hajeong Lee, Dong Ki Kim, Kwon Wook Joo, Yon Su Kim, Yong Chul Kim

**Affiliations:** 1 Department of Internal Medicine, Chung-Ang University Gwangmyeong Hospital, Gwangmyeong, Korea; 2 Department of Biomedical Sciences, Seoul National University College of Medicine, Seoul, Korea; 3 Department of Internal Medicine, Korea University Guro Hospital, Seoul, Korea; 4 Department of Internal Medicine, Hanyang University Guri Hospital, Guri, Korea; 5 Department of Internal Medicine, Hallym University Dongtan Sacred Heart Hospital, Hwaseong, Korea; 6 Department of Statistics and Actuarial Science, Soongsil University, Seoul, Korea; 7 Department of Internal Medicine, Seoul National University Hospital, Seoul, Korea; 8 Present Address: Department of Internal Medicine, Seoul National University College of Medicine, Seoul, Korea

**Keywords:** Kidney, Risk factors, Depression

## Abstract

Depression is associated with impaired quality of life and increased morbidity and mortality in end-stage kidney disease (ESKD) patients and kidney transplantation (KT) recipients. Depression incidence after KT is unclear. We compared depression incidence among KT recipients, ESKD patients, and healthy controls (HCs). We analyzed a nationwide health insurance database in South Korea and identified patients who underwent KT during 2007–2015. Participants were matched for age, sex, and inclusion year. KT and ESKD patients were further matched for hypertension and diabetes mellitus history. The incidence rate (IR, per 1000 patients-years) of depression was compared among KT, ESKD, and HC groups. We analyzed 5,234 patients per group. Depression incidence was markedly lower in KT than ESKD patients (IR, 18.87 vs. 58.03; hazard ratio [HR], 0.33; 95% confidence interval [CI], 0.30‒0.36), but only slightly higher in KT recipients than in HCs (IR, 18.87 vs. 17.49; HR, 1.08; 95% CI, 0.96‒1.22). After adjusting for comorbidities, the depression risk was lower in KT recipients than in HCs (adjusted HR, 0.52; 95% CI, 0.44‒0.62; *p* < 0.001), whereas it remained higher in ESKD patients than in HCs (adjusted HR, 1.60; 95% CI, 1.36‒1.87; *p* < 0.001). Among KT recipients, older age, female sex, lower economic status, and more comorbidities were associated with increased depression risk. Incident depression after KT increased mortality, graft failure, and death-censored graft failure risks in KT recipients. Our data suggest a broader role of KT than previously appreciated in terms of improving quality of life by reducing depression risk.

## Introduction

Globally, depression is a common mental disorder, and is characterized by persistent sadness and loss of interest in activities. One of the contributor to the non-fatal burden of disease globally^1^, depression is associated with substantial impairments in fulfilment of productive and social roles^2^.

Depression is more common in patients with chronic kidney disease (CKD) than in those without CKD^3^. Previous studies reported a prevalence of depression in CKD patients of about 21‒38%^3–6^. Depression is associated with an increased risk of morbidity, mortality and even progression to dialysis-dependent end-stage kidney disease (ESKD)^4,7,8^. As with other risk factors, it is necessary to explain how depression itself affects hard outcomes in ESKD patients. Possible mechanisms were suggested in several studies. Depression can induce non-adherence to proper diets, drugs, and medical service which can lead to malnutrition and decreased health status^9–11^. Also, in terms of cardiovascular diseases, there are reports that depression could affect biologic activities such as heart rate variability, inflammatory biomarker, and platelet function^12–15^.

Kidney transplantation (KT) is the only therapeutic option for patients with ESKD. In addition to the recovery of kidney function, KT facilitates improvement in emotional and psychological states and ultimately offers a significant advantage in survival^16^. One meta-analysis showed that depression increases risk mortality in organ transplantation including KT^17^. Some studies identified no significant differences in depressive symptoms between KT recipients and CKD patients^18,19^, but other studies have shown that transplantation could provide psychological improvements in KT recipients^20,21^. Despite several studies of mood disorder in KT and ESKD groups, it was difficult to find studies that accurately calculated and compared incidence of depression in those groups.

In this study, we aimed to explore the incidence of depression in KT recipients as compared with that in ESKD patients and healthy controls (HCs), and to determine the impact of depression in KT recipients.

## Materials and methods

### Ethical considerations

This study complied with the principles of the Declaration of Helsinki. The institutional review board of the Seoul National University Hospital (Institutional Review Board No. E-1904–111-1028) approved this study. The Ministry of Health and Welfare of South Korea approved to use the National Health Insurance Database (NHID). The need to obtain informed consent was waived because we used the data about study participants which were anonymized and de-identified.

### Data source

We reviewed nationwide population-based data from NHID of South Korea, from January 2007 to December 2015. In South Korea, the National Health Insurance developed a registration system based on the specific code for each rare or incurable disease to provide benefits for medical costs. KT and ESKD are included in this rare, incurable disease category. The subjects of this study were selected based on the International Classification of Disease, 10^th^ revision (ICD-10) code, rare or incurable disease code, prescription code, and procedure code. KT recipients who had received a KT from a living or deceased donor during the study period were selected based on the presence of a newly registered code for KT (V005) during the study period. The time of discharge was defined as the initiation of follow-up. ESKD patients were identified by the code for hemodialysis or peritoneal dialysis (Z49, Z99.2, V001, V003, O7011-7020, O7071-7075) for more than 3 months and who had never been registered with the code for KT (V005). Those who had not undergone either renal replacement therapy (RRT) or KT were classified by HCs. KT recipients were 1:1 matched with ESKD patients after adjustment for age, sex, inclusion year, the presence of hypertension and diabetes mellitus. Also, KT recipients were 1:1 matched with HCs after adjustment for age, sex, and inclusion year. Subjects were excluded based on the following criteria: (1) age less than 20 years, (2) previous diagnosis with depression before the start of this study, and (3) having received two or more organ transplantations.

### Data collection

Demographic characteristics, including age, sex, income level, and the type and duration of renal replacement therapy were collected from the baseline data in the NHID. Comorbidities, including hypertension, diabetes mellitus, dyslipidemia, were identified by the presence of ICD-10 codes or by two or more prescriptions of medications for hypertension, diabetes mellitus, and dyslipidemia within 1 year prior to the inclusion year. Charlson Comorbidity Index (CCI) score was calculated to assess the severity of the medical condition of the study subjects^22^. The history of immunosuppressants including desensitization, induction therapy, and maintenance were collected in KT recipients.

### Study outcomes

We compared the incidence of depression in the three groups. Depression was identified by the ICD-10 code (F32, F33) which was newly registered in the study period. Since the follow-up of incident depression was performed until December 2017, all subjects had a follow-up period of at least 1 year.

The secondary outcome was the long-term effect of incident depression in KT recipients in terms of all-cause mortality, graft failure, and death-censored graft failure. All-cause mortality was defined as death for any reason after KT. Graft failure included death for any reason after KT or dialysis-related code issued 1 month after KT. Among these, all other events, except death, were separately defined as death-censored graft failure.

### Statistical analysis

Continuous variables are presented as mean ± standard deviation, and categorical variables are expressed as percentages. We used the Kruskal–Wallis test for analysis of continuous variables. Chi-square test was used for analysis of categorical variables. The incidence rate (IR) of depression in the three groups was expressed by the number of events per 1000 person-years. Cox proportional hazard regression models were used to calculate the hazard ratio (HR) and 95% confidence interval (CI) for the risk of incident depression in KT recipients. The cumulative incidences of depression were compared among the three groups using the long-rank test. Time-dependent Cox regression analysis was used to evaluate the incidence of secondary outcomes, including all-cause mortality, graft failure, and death-censored graft failure. SAS 9.4 software (SAS Institute, Cary, NC, USA) was used to perform statistical analyses. Statistical significance was set at *p* < 0.05.

## Results

### Baseline characteristics

**Figure 1:**
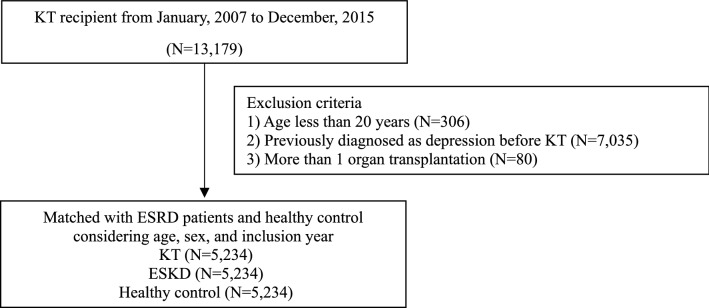
Flow chart.

From 2007 to 2015, we found that total 13,179 patients underwent KT in South Korea. After 1:1 matching, KT, ESKD and HC groups included 5234 matched subjects, respectively (Fig. 1). Table 1 shows the baseline characteristics of the study participants. The mean age was 43.68 ± 10.4, and 65.44% of the patients were male in all groups. The ESKD and KT groups had lower economic status than the HC group, and the ESKD group had a lower economic status than the KT group. The prevalence of hypertension, diabetes mellitus, and dyslipidemia in the ESKD and KT groups was 91%, 31%, and 39%, respectively. In HC group, 13.39%, 5.04%, and 9.30% of participants had hypertension, diabetes mellitus, and dyslipidemia, respectively, which were all lower than those in ESKD and KT groups.

**Table 1:** Baseline characteristics of study population.

	ESKD (N = 5234)	KT (N = 5234)	HC (N = 5234)	*p*-value
**Inclusion year**				1
2007	452 (8.64)	452 (8.64)	452 (8.64)	
2008	551 (10.53)	551 (10.53)	551 (10.53)	
2009	550 (10.51)	550 (10.51)	550 (10.51)	
2010	529 (10.11)	529 (10.11)	529 (10.11)	
2011	649 (12.40)	649 (12.40)	649 (12.40)	
2012	670 (12.80)	670 (12.80)	670 (12.80)	
2013	613 (11.71)	613 (11.71)	613 (11.71)	
2014	639 (12.21)	639 (12.21)	639 (12.21)	
2015	581 (11.10)	581 (11.10)	581 (11.10)	
**Age**	43.68 ± 10.4	43.68 ± 10.4	43.68 ± 10.4	1
20–29	528 (10.09)	528 (10.09)	528 (10.09)	
30–39	1319 (25.20)	1319 (25.20)	1319 (25.20)	
40–49	1746 (33.36)	1746 (33.36)	1746 (33.36)	
50–59	1319 (25.20)	1319 (25.20)	1319 (25.20)	
60–69	311 (5.94)	311 (5.94)	311 (5.94)	
≥ 70	11 (0.21)	11 (0.21)	11 (0.21)	
Male	3425 (65.44)	3425 (65.44)	3425 (65.44)	1
**Income**				< 0. 001
Aid	1109 (21.19)	731 (13.97)	99 (1.89)	
Q1	1496 (28.58)	1031 (19.70)	1382 (26.40)	
Q2	1125 (21.49)	1057 (20.19)	1302 (24.88)	
Q3	866 (16.55)	1094 (20.90)	1238 (23.65)	
Q4	638 (12.19)	1321 (25.24)	1213 (23.18)	
Hypertension	4778 (91.29)	4778 (91.29)	701 (13.39)	< 0.001
Diabetes mellitus	1672 (31.94)	1672 (31.94)	264 (5.04)	< 0.001
Dyslipidemia	2086 (39.85)	2813 (53.74)	487 (9.30)	< 0.001
**RRT type**				< 0.001
None	0 (0)	1878 (35.88)	5234 (100)	
HD	3856 (73.67)	2192 (41.88)	0 (0)	
PD	1151 (21.99)	892 (17.04)	0 (0)	
Mixed	227 (4.34)	272 (5.20)	0 (0)	
RRT duration ≥ 1 year	3091 (59.06)	2640 (50.44)	0 (0)	< 0.001
Desensitization	0 (0)	732 (13.99)	0 (0)	< 0.001
**Induction**				< 0.001
No use	5234 (100)	336 (6.42)	5234 (100)	
ATG	0 (0)	353 (6.74)	0 (0)	
Basiliximab	0 (0)	4545 (86.84)	0 (0)	
**Maintenance**				< 0.001
None	5234 (100)	143 (2.73)	5234 (100)	
Tacrolimus	0 (0)	4178 (79.82)	0 (0)	
Cyclosporin	0 (0)	913 (17.44)	0 (0)	

Data are presented as the mean ± standard deviation, or n (%).

*ESKD* end-stage kidney disease, *KT* kidney transplantation, *HC* healthy control, *RRT* renal replacement therapy, *HD* hemodialysis, *PD* peritoneal dialysis, *ATG* antithymocyte globulin.

In the ESKD group, 73.67% received hemodialysis, 21.99% received peritoneal dialysis, and 4.34% were reported that both hemodialysis and peritoneal dialysis were performed. Among KT recipients, 41.88% underwent hemodialysis, 17.04% underwent peritoneal dialysis, and 35.88% had been never treated with any dialysis treatment. More than half of the ESKD and KT groups received dialysis treatment for more than a year. In the KT groups, desensitization treatment was performed in 13.99% before transplantation. Additionally, 93.58% of KT recipients received induction immunosuppressive therapy with antithymocyte globulin or basiliximab. Maintenance immunosuppressive therapy was administered to almost all KT recipients.

### Incidence of depression

As shown in Tables 2 and 3, we analyzed the IR per 1000 person-years of incident depression in the three groups. KT recipients had a markedly lower incidence of depression during follow-up period than ESKD patients (IR, 18.87 vs. 58.03 per 1000 person-years; HR, 0.33; 95% CI, 0.30‒0.37; *p* < 0.001) after adjusting for age, sex, income level, presence of hypertension, diabetes mellitus, and dyslipidemia, CCI score, and RRT duration.

**Table 2:** Incidence rate and risk of depression in ESKD and KT groups.

	Total N	Incident depression	IR*	Model 1**		Model 2***	
				HR (95% CI)	*p*-value	HR (95% CI)	*p*-value
ESKD	5234	1373	58.03	1 (reference)	< 0.001	1 (reference)	< 0.001
KT	5234	568	18.87	0.33 (0.30, 0.36)		0.33 (0.30, 0.37)	

*IR: incidence rate per 1000 person-years.

**Model 1 was adjusted for age and sex.

***Model 2 was adjusted for age, sex, income level, presence of hypertension, diabetes mellitus and dyslipidemia, CCI score, and RRT duration.

*ESKD* end-stage kidney disease, *KT* kidney transplantation.

**Table 3:** Incidence rate and risk of depression in ESKD, KT and HC groups.

	Total N	Incident depression	IR*	Model 1**		Model 2***	
				HR (95% CI)	*p*-value	HR (95% CI)	*p*-value
ESKD	5234	1373	58.03	3.34 (3.02, 3.69)	< 0.001	1.60 (1.36, 1.87)	< 0.001
KT	5234	568	18.87	1.08 (0.96, 1.22)	0.198	0.52 (0.44, 0.62)	< 0.001
HC	5234	541	17.49	1 (reference)		1 (reference)	

*IR: incidence rate per 1000 person-years.

**Model 1 was adjusted for age and sex.

***Model 2 was adjusted for age, sex, presence of hypertension, diabetes mellitus and dyslipidemia, and CCI score.

*ESKD* end-stage kidney disease, *KT* kidney transplantation, *HC* healthy control.

**Figure 2:**
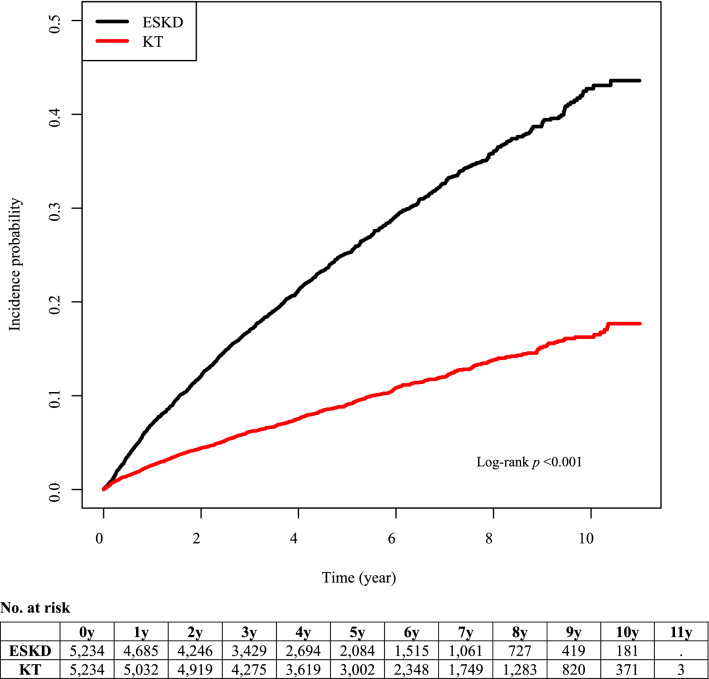
Cumulative risk for incident depression in KT and ESKD group. Abbreviation: ESKD, end-stage kidney disease; KT, kidney transplant.

**Figure 3:**
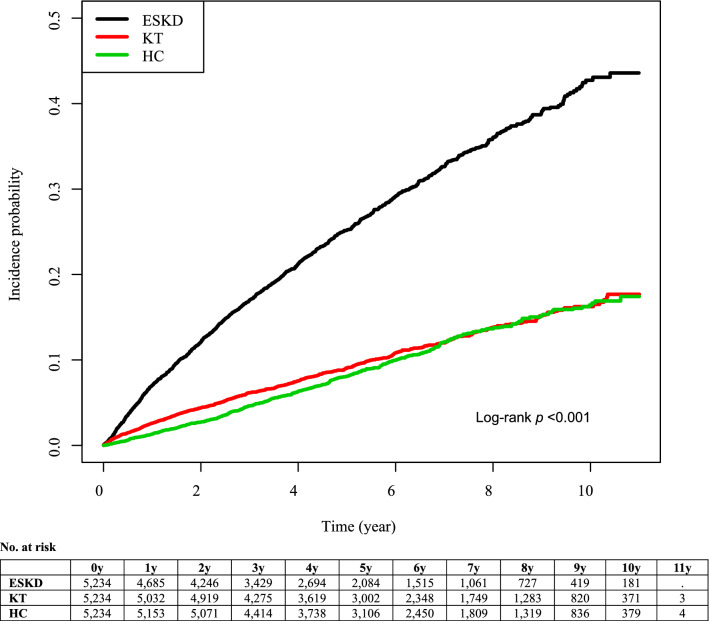
Cumulative risk for incident depression in KT, ESKD and HC group. Abbreviation: ESKD, end-stage kidney disease; KT, kidney transplant; HC, healthy control.

Interestingly, after adjusting for comorbidity status, including hypertension, diabetes mellitus, and CCI score, KT recipients showed a lower risk of incident depression than HCs (adjusted HR, 0.52; 95% CI, 0.44‒0.62; *p* < 0.001). On the other hand, risk of incident depression in ESKD patients remained at a higher risk compared with HCs (adjusted HR, 1.60; 95% CI, 1.36‒1.87, *p* < 0.001). The cumulative risk for incident depression in the KT, ESKD, and HC groups are shown in Figs. 2 and 3.

### Clinical characteristics of incident depression after KT

The differences in clinical characteristics according to the incidence of depression in KT recipients are shown in Table 4. In the KT group, KT recipients with depression showed a higher proportion of women, lower economic status, and higher prevalence of diabetes mellitus than those without depression. There was no statistically significant difference between the two groups according to the type and duration of RRT and immunosuppressant therapy.

**Table 4:** Clinical characteristics according to the presence of incident depression in KT group.

	Depression		*p*-value
	No (N = 4666)	Yes (N = 568)	
**Age**			0.007
20–29	482 (10.33)	46 (8.10)	
30–39	1204 (25.80)	115 (20.25)	
40–49	1550 (33.22)	196 (34.51)	
50–59	1151 (24.67)	168 (29.58)	
60–69	269 (5.77)	42 (7.39)	
≥ 70	10 (0.21)	1 (0.18)	
Male	3088 (66.18)	337 (59.33)	0.001
**Income**			< 0.001
Aid	594 (12.73)	137 (24.12)	
Q1	925 (19.82)	106 (18.66)	
Q2	962 (20.62)	95 (16.73)	
Q3	986 (21.13)	108 (19.01)	
Q4	1199 (25.70)	122 (21.48)	
Hypertension	4265 (91.41)	513 (90.32)	0.385
Diabetes mellitus	1458 (31.25)	214 (37.68)	0.002
Dyslipidemia	2498 (53.54)	315 (55.46)	0.386
**RRT type**			0.079
None	1686 (36.13)	192 (33.80)	
HD	1947 (41.73)	245 (43.13)	
PD	781 (16.74)	111 (19.54)	
Mixed	252 (5.40)	20 (3.52)	
RRT duration ≥ 1 year	2345 (50.26)	295 (51.94)	0.450
Desensitization	662 (14.19)	70 (12.32)	0.227
**Induction**			0.112
No use	290 (6.22)	46 (8.10)	
ATG	322 (6.90)	31 (5.46)	
Basiliximab	4054 (86.88)	491 (86.44)	
**Maintenance**			0.348
None	131 (2.81)	12 (2.11)	
Tacrolimus	3731 (79.96)	447 (78.70)	
Cyclosporin	804 (17.23)	109 (19.19)	

Data are presented as the mean ± standard deviation, or n (%).

*KT* kidney transplant, *CCI* Charlson Comorbidity Index, *RRT* renal replacement therapy, *HD* hemodialysis, *PD* peritoneal dialysis, *ATG* antithymocyte globulin**.**

In Table 5, we found that age > 60 years, female sex, lower economic status, and higher CCI score were risk factors for incident depression after KT. The type and duration of RRT before transplantation, desensitization, and type of immunosuppressant use were not statistically significant risk factors for the occurrence of depression.

**Table 5:** Predictive risk factors for incident depression in KT group.

	Univariate		Multivariate	
	HR (95% CI)	*p*-value	HR (95% CI)	*p*-value
**Age**		< 0.001		< 0.001
20–29	0.56 (0.41, 0.78)		0.60 (0.43, 0.84)	
30–39	0.59 (0.47 ,0.75)		0.58 (0.45, 0.74)	
40–49	0.80 (0.65, 0.98)		0.76 (0.62, 0.94)	
50–59	1 (reference)		1 (reference)	
60–69	1.15 (0.82, 1.61)		1.21 (0.86, 1.70)	
≥ 70	1.01 (0.14, 7.21)		1.25 (0.17, 8.93)	
**Sex**		0.009		0.002
Male	1 (reference)		1 (reference)	
Female	1.25 (1.06, 1.48)		1.32 (1.11, 1.56)	
**Income**		< 0.001		< 0.001
Aid	1 (reference)		1 (reference)	
Q1	0.56 (0.44, 0.73)		0.54 (0.41, 0.70)	
Q2	0.48 (0.37, 0.63)		0.45 (0.35, 0.59)	
Q3	0.53 (0.41, 0.69)		0.50 (0.39, 0.65)	
Q4	0.50 (0.39, 0.64)		0.43 (0.33, 0.56)	
**CCI score**		< 0.001		0.004
1–2	1 (reference)		1 (reference)	
3–4	1.21 (0.90, 1.63)		1.17 (0.87, 1.58)	
≥ 5	1.73 (1.30, 2.30)		1.56 (1.16, 2.10)	
Hypertension	0.90 (0.69, 1.19)	0.477	1.01 (0.76, 1.35)	0.927
Diabetes mellitus	1.35 (1.14, 1.60)	0.001	1.08 (0.90, 1.31)	0.401
Dyslipidemia	1.12 (0.95, 1.32)	0.178	1.11 (0.93, 1.31)	0.251
**RRT type**		0.101		0.254
None	0.87 (0.72, 1.05)		0.87 (0.67, 1.14)	
HD	1 (reference)		1 (reference)	
PD	1.09 (0.87, 1.37)		1.10 (0.87, 1.38)	
Mixed	0.70 (0.45, 1.11)		0.74 (0.47, 1.17)	
RRT duration ≥ 1 year	1.12 (0.95, 1.32)	0.168	0.92 (0.72, 1.19)	0.527
Desensitization	1.05 (0.82, 1.35)	0.715	1.05 (0.81, 1.36)	0.696
**Induction**		0.985		0.772
No use	0.99 (0.63, 1.57)		1.10 (0.68, 1.78)	
ATG	1 (reference)		1 (reference)	
Basiliximab	1.02 (0.71, 1.46)		0.98 (0.68, 1.41)	
**Maintenance**		0.581		0.619
None	0.83(0.47, 1.47)		0.88 (0.49, 1.59)	
Tacrolimus	1 (reference)		1 (reference)	
Cyclosporin	0.91(0.74, 1.12)		0.90 (0.72, 1.12)	

*KT* kidney transplantation, *CCI* Charlson Comorbidity Index, *RRT* renal replacement therapy, *HD* hemodialysis, *PD* peritoneal dialysis, *ATG* antithymocyte globulin.

### Long-term effect of incident depression after KT

**Figure 4:**
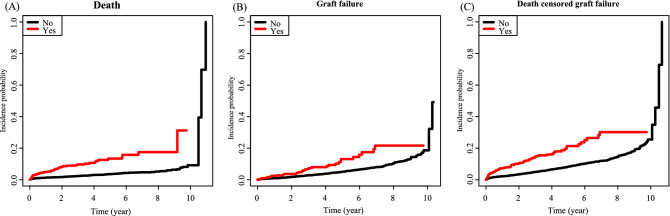
Cumulative risk for death, graft failure, and death censored graft failure according to presence of incident depression in KT group ((**A**): death, (**B**): graft failure, (**C**): death censored graft failure).

Table 6 and Fig. 4 show the long-term effects of incident depression as death, graft failure, and death-censored graft failure in KT recipients. Incident depression after KT was found to increase the risk of death (HR, 4.46; 95% CI, 3.24‒6.13, *p* < 0.001), graft failure (HR, 2.57; 95% CI, 1.99‒3.31, *p* < 0.001), and death-censored graft failure (HR, 2.10; 95% CI, 1.48‒2.99, *p* < 0.001).

**Table 6:** Risk of death, graft failure and death-censored graft failure according to the presence of depression in KT group.

Depression	N	Event	HR (95% CI)	*p*-value
**Death**				
No	5234	195	1 (reference)	< 0.001
Yes	568	49	4.46 (3.24, 6.13)	
**Graft failure**				
No	5234	489	1 (reference)	< 0.001
Yes	515	69	2.57 (1.99, 3.31)	
**DCGF**				
No	5234	312	1 (reference)	< 0.001
Yes	515	35	2.10 (1.48, 2.99)	

*KT* kidney transplantation, *DCGF* death-censored graft failure.

## Discussion

This nationwide population-based study aimed to evaluate the incidence of newly diagnosed depression in KT recipients compared with ESKD and HC groups. We found that the incidence of depression in the KT group was lower than that in the ESKD group, and unexpectedly, it was lower than that in the HC group after adjustment for comorbidities. Furthermore, we confirmed that incident depression adversely affected mortality and graft outcomes in KT recipients.

Depression is one of the most important public health issues^23,24^. Persons with a personal or family history of psychiatric disorders, disability, and overall medical burden, particularly that of chronic diseases, are more susceptible to depression^25,26^. Depression is more common in patients with CKD than in the general population^3,27^. Depression is a common comorbidity in CKD patients, and it is associated with an increased risk of malnutrition, hospital admission and even early initiation of RRT^3,9,10^. In our study, ESKD patients showed a higher incidence of depression than HCs, even after adjustment for age, sex and comorbidities.

KT improves not only overall survival, but also quality of life in patients with ESKD^16^. Despite such advantages, KT recipients are challenged with rapid physical, psychological, and social changes^5,28–30^. Several studies have investigated the effect of KT on psychological aspects, and have found controversial results. Some studies showed that psychological distress of KT recipients did not seem to improve after KT, as compared with ESKD patients^18,19,31^. Other studies reported that KT was superior in relieving emotional distress and improving psychological well-being over hemodialysis or peritoneal dialysis in ESKD patients^32–36^. In a meta-analysis, the prevalence of depression, according to self-report or clinician-rated tools, was 26.6% in KT recipients and 39.3% in ESKD patients^27^. Old age^37–41^, female sex^37,39–41^, white race^37^, unemployment status and a poor financial situation^16,38,39,42^, and a low level of education^39,40^ are reported to increase the risk of depression in KT recipients. In terms of kidney function, KT recipients with a longer functional graft showed lower stages of depression^33^.

During our study period, among 5234 KT recipients, 568 subjects experienced incident depression after KT. The incidence was significantly lower than that in patients with ESKD, and was even lower than that in HC group after considering age, sex, and comorbidities. Based on this result, KT has psychological benefits beyond the recovery of kidney function. As with the results of many precious studies, several factors were associated with the risk of incident depression in KT recipients. Age > 60 years, female sex, poorer economic status, and higher CCI score could be predictive risk factors for depression in KT recipients. However, the type and duration of RRT and type of immunosuppressant were not related to an increased risk of depression.

We analyzed the long-term effect of incident depression after KT on all-cause mortality, graft failure, and death-censored graft failure. A prospective cohort study showed that depressive symptoms were not significantly associated with medical outcomes^43^. In contrast, many studies including meta-analysis found that depression may affect the disease process and graft survival^17,30,44^. Our results also showed a higher incidence of all-cause mortality, graft failure, and death-censored graft failure in group with depression than in the group without depression in KT recipients through time-dependent Cox analysis. For KT recipients, it is very important to take immunosuppressive medication regularly and undergo regular examinations, including blood and urine tests and imaging. Depressive mood can reduce the patient’s adherence to medication and hospital visits and can discourage patients from modifying their lifestyle. Furthermore, the lack of social support due to the disconnection of relationships should also be considered.

There were several limitations to this study. Although this study used national health insurance data with a larger number of subjects than previous studies, this was a retrospective cohort study based on diagnosis and prescription history. Second, the assessment of depression was determined only by the ICD-10 code, and other methods, such as questionnaires and psychological evaluations, were not considered. Unfortunately, it was difficult to apply such a method to studies using national health insurance data. Third, due to the limited method for detecting the incident depression, the degree of depression was not considered in this study. If the degree of depression was discriminative, it would have been possible to compare the degree of depression for each group. Fourth, only depression was analyzed among several mental problems that could occur after KT. In future research, we will investigate the incidence of sleep disorders, eating disorders, anxiety, and neurological disorders, such as dementia, and their relationship with prognosis. Fifth, we could not review systemic corticosteroid therapy in all groups in this study. Corticosteroid is well-known drugs that have various side effects including psychiatric effect. We hope that further study must consider the corticosteroid as a variable in the evaluation of the incident depression. Sixth, we defined HCs as the absence of renal replacement therapy (RRT) or kidney transplantation (KT). HCs may include CKD patients as well as hypertension and diabetes, which may be a bias that effected the incidence of depression.

In conclusion, KT recipients showed a markedly lower risk of incident depression than ESKD patients and even matched HCs after adjustment for comorbidities. There was a negative association incident depression on mortality and graft outcomes. If further research shows that the treatment of depression in KT recipients can improve long-term renal outcome, the implications of this study will be further strengthened. Attention to the psychological aspects of KT recipients is important when managing patients undergoing KT. KT has a broader role than originally conceived. Moreover, the management of depression in patients with ESKD and KT recipients may have a significant impact on the patient’s prognosis.

## Data Availability

The datasets generated and/or analyzed in the current study are available from the corresponding author on reasonable request.

## Abbreviations

CCI: Charlson comorbidity index
CI: confidence interval
CKD: chronic kidney disease
ESKD: end-stage kidney disease
HC: healthy controls
HR: hazard ratio
ICD-10: International Classification of Disease, 10th revision
IR: incidence rate
KT: kidney transplantation
